# Differential Patterns of Brain and Body Aging on MR Imaging

**DOI:** 10.1002/alz.093387

**Published:** 2025-01-09

**Authors:** Cyrus A. Raji, Somayeh Meysami, Sam Hashemi, Saurabh Garg, Nasrin Akbari, Ahmed Gouda, Yosef Gavriel Chodakiewitz, Thanh Duc Nguyen, Kellyann Niotis, David A. Merrill, Rajpaul Attariwala

**Affiliations:** ^1^ Mallinckrodt Institute of Radiology, Washington University in St. Louis, St. Louis, MO USA; ^2^ Pacific Brain Health Center, Pacific Neuroscience Institute and Foundation, Santa Monica, CA USA; ^3^ Saint John's Cancer Institute at Providence Saint John's Health Center, Santa Monica, CA USA; ^4^ Prenuvo, Vancouver, BC Canada; ^5^ Voxelwise Imaging Technology, Vancouver, BC Canada; ^6^ Vigilance Health Imaging Network Inc., Vancouver, BC Canada; ^7^ Early Medical, Austin, TX USA; ^8^ The Institute for Neurodegenerative Diseases (IND) Florida, Boca Raton, FL USA; ^9^ David Geffen School of Medicine at UCLA, Los Angeles, CA USA; ^10^ AIM Medical Imaging, Vancouver, BC Canada

## Abstract

**Background:**

Comparative information on how whole‐body organs are linked with age and the brain is lacking.

**Method:**

Overall, 7,149 healthy participants from four sites (Mean age 53.06 ± 12.95 years, 18‐97 years; 48% women; 52% men; 38% non‐white) were scanned on 1.5T MR machines with a whole‐body protocol. Whole body sequences utilized in the quantitative analyses were coronal T1 for organ, fat and muscle segmentation. Deep learning with FastSurfer on MPRAGE trained on 134 participants aged 27‐66 and segmented 96 brain regions. Partial correlation analysis was done controlling for sex and total intracranial volume for brain regions and total abdominal organ volume for abdominal organs. Multiple comparisons were accounted for using the Bonferroni method.

**Result:**

Age negatively correlated with gray matter (r_p_ = ‐0.38, p = 6.31×10^−244^) and white matter (r_p_ = ‐0.263, p = 4.03×10^−112^). Age positively correlated to cerebral ventricle volume (r_p_ = 0.493, p < .001). Increasing age was also inversely correlated with the lobar structural volumes: frontal lobe (r_p =_ ‐0.420 p = 2.70×10^−302^), temporal lobe (r_p =_ ‐0.378 p = 4.50×10^−240^), parietal lobe (r_p_ ‐0.373 with p = 2.22×10^−233^), occipital lobe (r_p =_ ‐0.259; p = 1.58×10^−108^). Age negatively correlated with AD risk regions: hippocampus (r_p_ = ‐0.288 and p = 9.79×10^−136^), posterior cingulate (r_p =_ ‐0.338, p = 4.37×10^−189^), precuneus (r_p =_ ‐0.321, p= 1.44×10^−169^). Beyond the brain, the kidney and psoas muscle showed significant negative correlations with age (kidney; r_p_= ‐0.114, p = 1.23×10^−211^) and the psoas muscle (r_p_ = ‐0.353, p= 1.13×10^−208^). Strikingly, visceral fat (vfat) was found to have a strong positive correlation with age, (r_p_ = 0.416, p= 8.81×10^−297^). Subcutaneous fat (sfat), by contrast, showed a null correlation (r_p_ = ‐0.01, p=1.0). Increasing age showed negative correlations with liver volumes (r_p_ = ‐0.226, p = 2.21×10^‐82^), spleen (r_p_ = ‐0.262, 5.46×10^‐112^) and total muscle volume (r_p_ = ‐0.317, 3.17×10^‐165^).

**Conclusion:**

Differential patterns of brain and body organ aging may lend insight into how age can increase the risk for common brain disorders in the elderly such as Alzheimer’s.

